# Neurocognitive Trajectories After 72 Weeks of First-Line Anti-retroviral Therapy in Vietnamese Adults With HIV-HCV Co-infection

**DOI:** 10.3389/fneur.2021.602263

**Published:** 2021-03-12

**Authors:** Robert H. Paul, Cecilia M. Shikuma, Nguyen Van Vinh Chau, Lishomwa C. Ndhlovu, Nguyen Tat Thanh, Andrew C. Belden, Dominic C. Chow, Glen M. Chew, Thomas A. Premeaux, Vo Trieu Ly, Joseph A. D. McBride, Jacob D. Bolzenius, Thuy Le

**Affiliations:** ^1^University of Missouri–St. Louis, St. Louis, MO, United States; ^2^Hawai'i Center for AIDS, University of Hawai'i at Manoa, Honolulu, HI, United States; ^3^Hospital for Tropical Diseases, Ho Chi Minh City, Vietnam; ^4^Cornell University School of Medicine, New York City, NY, United States; ^5^Oxford University Clinical Research Unit, Ho Chi Minh City, Vietnam; ^6^University of Medicine and Pharmacy at Ho Chi Minh City, Vietnam; ^7^Duke University School of Medicine, Durham, NC, United States

**Keywords:** human immunodeficiency virus, HCV (hepatitis C), co-infection, neurocognition, treatment

## Abstract

**Background:** Long-term neurocognitive outcomes following first-line suppressive anti-retroviral therapy (ART) remain uncertain for individuals with HIV and hepatitis C (HCV) co-infection. The study examined neurocognitive performance before and after 72 weeks of ART using repeated multivariate analyses and latent trajectory models.

**Methods:** One hundred and sixty adults with chronic, untreated HIV infection (*n* = 80 with HCV co-infection and *n* = 80 HIV mono-infected) and 80 demographically similar healthy controls were recruited from the Hospital for Tropical Diseases in Ho Chi Minh City and the surrounding community, respectively. Neurocognitive measures (adapted for use in Vietnam) and liver enzyme tests were compared across groups at baseline. Repeated multivariate and group-based trajectory analyses (GBTA) examined neurocognitive subgroup profiles of the co-infected individuals after 72 weeks of *de novo* efavirenz- (*n* = 41) or raltegravir-based (*n* = 39) ART.

**Results:** Baseline analyses revealed worse motor function in HIV-HCV co-infected individuals compared to both comparison groups. Longitudinal analyses revealed improved neurocognitive performance by week 48 for most participants regardless of treatment arm. GBTA identified a subgroup (35% of HIV-HCV sample) with persistent motor impairment despite otherwise successful ART. Higher HIV viral load and lower CD4^+^ T cell count at baseline predicted persistent motor dysfunction. Liver indices and ART regimen did not predict neurocognitive outcomes in HIV-HCV co-infected individuals.

**Conclusions:** Most HIV-HCV co-infected individuals achieve normative neurocognitive performance after 48 weeks of de novo suppressive ART. However, individuals with more severe HIV disease prior to ART exhibited motor impairment at baseline and 72 weeks after otherwise successful treatment. Interventions aimed at improving motor symptoms at the time of HIV treatment onset may improve long-term clinical outcomes in HIV-HCV co-infected adults.

## Introduction

As of 2016, 2.3 million cases of HIV-HCV co-infection were reported globally (1). HIV-HCV co-infection disproportionately affects individuals residing in resource-limited settings, where access to direct acting anti-viral treatment for HCV is less readily available (2–5). Recent studies report a high rate of HCV infection among PLWH in Vietnam, with a prevalence of nearly 90% in high-risk subgroups (6, 7). The individual and societal burden of co-occurring HIV and HCV infection is significant. Despite long-term use of anti-retroviral therapy (ART) HIV-HCV co-infected adults report increased use of medical services and level of disability, as well as a greater burden of chronic health complications compared to individuals with HIV mono-infection (8, 9).

HIV-HCV co-infection significantly increases risk for chronic neurocognitive difficulties. Additive or synergistic effects of co-infection have been observed most consistently in the domains of motor, executive function, and learning, with less involvement of core language (e.g., comprehension, reception, repetition), and memory retention abilities (10–14). While mechanisms underlying more neurocognitive difficulties remain unclear, preliminary evidence points toward HCV-associated liver dysfunction as an important contributing factor, particularly in the context of poorly controlled HIV (13, 14). Clifford et al. (11) reported no difference in neurocognitive profiles of HIV-HCV co-infected compared to HIV mono-infected individuals with stable HIV suppression. However, other studies report higher levels of activated CD4^+^ and CD8^+^ T cells (15), immune activation (16), and T cell exhaustion (15, 17) in co-infected individuals on stable ART. Additionally, Fabiani et al. (18) reported a significant association between global neurocognitive status and plasma levels of HCV-RNA in co-infected individuals who were receiving suppressive ART. These results suggest that HIV treatment alone does not prevent the development of neurocognitive complications, but longitudinal studies are needed to tease apart neurocognitive profiles of HIV presenting with or without comorbid HCV.

Few studies have examined neurocognitive profiles of co-infected individuals before and after the initiation of suppressive ART. To date, most studies have been cross-sectional in nature and comprised of participants with variable degrees of ART adherence and HIV viral suppression. Additionally, prior work has relied on traditional analytic methods that assume similar rates of neurocognitive sequelae across all co-infected individuals, rather than specific risk subgroups. Analytic methods such as group-based trajectory analysis [GBTA (19)] are sensitive to latent risk phenotypes that differ according to specific underlying mechanisms and have potential to inform novel therapeutic strategies to manage neurocognitive symptoms in this population.

In this study we used multivariate analysis of variance (MANOVA) tests to examine baseline differences on neurocognitive performance (per domain) between the three groups (HIV-HCV, HIV+, and HIV–) at their baseline assessment. Next, we conducted a repeated measures analysis of variance (RM-ANOVA) using data from weeks 0, 24, 48, and 72 within the HIV-HCV group to examine differences in neurocognitive testing performance over time and as a function of treatment arm (i.e., raltegravir vs. efavirenz). Finally, we conducted an exploratory group-based trajectory analysis within the HIV co-infected group to test for latent class groups of individuals following similar neurocognitive trajectories over time and agnostic to treatment arm. We then examined whether belonging to specific neurocognitive latent class trajectories differed in relation to treatment arm, liver function, or HIV disease indices. We hypothesized that viral, immune, and liver indices would predict neurocognitive performance at baseline (pre-ART) and differentiate individual neurocognitive trajectories from baseline through week 72 post-ART.

## Materials and Methods

### Statement of Ethics

The study was approved by the ethics committees of the Vietnam Ministry of Health, the Hospital for Tropical Diseases (HTD) in Ho Chi Minh City (HCMC), University of Hawaii at Manoa in the U.S., and the Oxford University Tropical Research Ethics Committee in the U.K.

### Study Participants

160 PLWH (80 with HIV-HCV co-infection) were recruited from the Hospital for Tropical Diseases in Ho Chi Minh City. All participants with HIV reported no history of ART. The HIV-HCV co-infected participants were enrolled in a phase IV randomized, controlled trial comparing raltegravir- vs. efavirenz-based ART on clinical outcomes over 72 weeks (ClinicalTrials.gov Identifier: NCT01147107). Inclusion criteria included: (1) age 18–65; (2) education level > 6 years; (3) able and willing to provide written informed consent; (4) laboratory confirmed HIV; (5) no prior use of ART; and (6) eligible to initiate ART based on guidelines from the Vietnam Ministry of Health during study enrollment from 2014 to 2016 (i.e., CD4^+^ T cell count <350 and/or WHO stage III or IV).

Additional inclusion criteria for HIV-HCV co-infected participants included detectable serum HCV RNA, serum aspartate aminotransferase (AST), alanine aminotransferase (ALT) ≤80 U/L, and creatinine clearance ≥60 mL/min. Exclusion criteria included: (1) history of anti-viral treatment for HIV or HCV; (2) positive hepatitis B surface antigen; (3) clinical evidence of de-compensated cirrhosis (e.g., ascites, encephalopathy, esophageal bleeding); (4) history of AIDS-defining illness within the preceding 2 weeks from study entry; and/or (5) possible pregnancy or intent to breastfeed during the study period.

Demographically similar healthy controls (*n* = 80) were recruited from the surrounding community. Inclusion and exclusion criteria were similar between the control group and the two HIV-infected groups, with the exception of HIV and HCV seronegative status. Study participants provided written informed consent following a thorough explanation of study procedures.

### HIV Treatment

Following the baseline evaluation, HIV-HCV co-infected participants were randomized to initiate *de novo* ART comprised of emtricitabine and tenofovir combined with either raltegravir (*n* = 39) or efavirenz (*n* = 41). The primary endpoint for the clinical trial was the frequency of AST or ALT toxicities grade >2. The secondary endpoints were HIV RNA <150 copies/mL, change in CD4^+^ counts, time to AIDS or death, and neurocognitive function. Preliminary analyses over 72 weeks revealed higher rates of hepatotoxicity and a higher number of total adverse events in the efavirenz arm compared to raltegravir arm, but HIV viral suppression was similar in both groups (20).

### Neurocognitive Assessment

Neurocognitive evaluations were completed at baseline for all three groups and at follow-up for the HIV-HCV co-infected participants. The test battery was selected based on sensitivity to both HIV and HCV mono-infection as described in prior studies (10, 14, 21), Prior to enrollment, the neurocognitive tests were reviewed for cultural relevance by a consensus panel comprised of local stakeholders in Vietnam (e.g., hospital staff and study personnel) and US investigators. The panel recommended modifications to the verbal learning and memory test and lexical fluency. Modifications to the verbal learning and memory test included replacement of English words with Vietnamese words matched on content meaning, level of familiarity among the target population, degree of abstraction/concreteness, and phonological complexity. Lexical fluency was modified by replacing fluency for English letters (F, A, S) with fluency for first names.

The test battery included the following cognitive domains (see Supplementary Table 1): *Psychomotor Speed:* Color Trails 1 (22), and Digit-Symbol (23); *Executive Function:* Digit Span Backward, Color Trails 2 (22), Action Fluency (24), and fluency for first names (25); *Learning and Memory:* total recall on the learning and delayed trials of the Hopkins Verbal Learning Test-Revised [HVLT-R (26)], and Brief Visuospatial Memory Test-Revised [BVMT-R (27)]; *Motor:* Grooved Pegboard dominant and non-dominant hand (28) and gait measured vis-à-vis time to complete a 10-meter midline cross-over walk (29), averaged across three trials.

Alternate test forms were utilized when applicable. Performances on the neurocognitive tests were converted to standardized scores using regression-based norms developed from the uninfected group. Raw scores were utilized as dependent variables in the baseline multivariate analyses, and regression-based standardized scores were utilized to determine the frequency of clinically relevant impairment, defined as *z* below −1.0.

### Mood Assessment

Participants completed the Beck Depression Scale-II [BDI-II (30)], which had been translated into Vietnamese in prior work (31). Total score served as the dependent variable.

### Viral and Immunological Indices

CD4^+^ T cell count, HIV viral load, and HCV viral load (co-infected participants) were performed in real time at the HTD for the HIV-HCV subgroup.

### Liver Function

Liver function testing was conducted for HIV-HCV co-infected participants. Dependent variables included serum levels of ALT, AST, alpha-fetoprotein (AFP), and transient liver elastography using FibroScan® (Fibrometer).

### Statistical Analysis

Data were analyzed using IBM SPSS 26.0 (Armonk, New York), STATA 14.2 (College Station, Texas), and STATA plugin for estimating group-based trajectory models (32). Preliminary analyses examined missingness, presence of outliers, and non-normal distributions. Age, number of years of education, distribution of sex, and self-reported depression on the BDI-II were compared between the three groups to identify covariates for inclusion in the baseline analyses. Results revealed higher BDI-II scores among co-infected and HIV mono-infected participants compared to healthy controls and higher education among the control group compared to the HIV-infected participants. BDI-II scores were included as a covariate in all multivariate analyses. Education was included in the regression-based standardized scores.

Baseline neurocognitive performances were compared using a series of MANOVAs and MANCOVAs with group (HIV-HCV co-infected, HIV mono-infected, uninfected healthy controls) serving as the independent variable, neurocognitive performance on tests that were grouped by domain (see Table 1) as the dependent variables, and BDI-II total score as the covariate. ANOVA tests were used for univariate *post-hoc* comparisons, and False Discovery Rate [FDR (33)] was applied to adjust for multiple comparisons. The percentages of individuals in each group with neurocognitive test performance worse than −1.0 (z score) were compared using Chi Square test. All *p*-values were considered significant at 0.05 (two sided).

**Table 1:** Baseline demographic and clinical characteristics.

	**HIV-HCV (*n* = 80)**	**HIV(*n* = 80)**	**Controls (*n* = 80)**	**p-value**
Sex (male)	68 (87%)	69 (86%)	62 (78%)	0.191
Age (years)	32 (31–36)	32 (27–37)	31 (24–44)	0.945
Plasma CD4 (cells/μL)	118 (27–280)	–	–	–
Plasma HIV viral load (log10 copies/mL)	5.2 (4.9–5.5)	–	–	
Plasma HCV viral load (log10 copies/mL)	6.6 (5.7–7.2)	–	–	–
Beck Depression Inventory-II	16 (14)	14.50 (12)	7.00 (10)	**<0.0001**
Education				0.437
Secondary school	63 (80.8%)	68 (85.0%)	64 (80.0%)	
High school	8 (10.3%)	7 (8.8%)	13 (16.3%)	
College and above	7 (9.0%)	5 (6.3%)	3 (3.8%)	

*Summary statistic is absolute count (%) for categorical variables and median (IQR) for continuous data. Bold values represent statistically significant differences between groups*.

Neurocognitive performances from weeks 0, 24, 48, and 72 were examined for the co-infected group using RM-ANOVA tests. Treatment arm served as the independent variable and *z*-scored neurocognitive test scores served as the dependent variable. GBTA (19) was used to explore the possibility of latent classes existing within the HIV-HCV subgroup who followed distinct neurocognitive trajectories across the 72-week observation period. The best fit trajectory group number and shape were determined using Bayesian Information Criterion (34). Posterior probabilities and odds ratios were used to assess overall model fit parameters (35) and are reported in Supplementary Materials. Group homogeneity was determined by minimum fit requirements of group average posterior probability >0.7 and odds of correct classification >4.0 (36). Individuals were assigned to the group for which posterior probability of membership was highest. Baseline disease variables, immune indices, ART regimen, liver function test results, demographic variables, and psychosocial factors were tested as possible predictors of latent class trajectory subgroups using multiple linear and logistic regressions.

## Results

### Sample Characteristics

Demographic characteristics are provided in Table 1. Analyses of baseline demographic data revealed no differences in age or the proportion of males to females. As noted above, the HIV-infected groups reported a lower average number of years of education than the uninfected group (~1-year difference between groups). Significant differences were also observed on the BDI-II, with higher scores reported by co-infected and HIV mono-infected individuals compared to uninfected controls. As expected, co-infected individuals randomized to the raltegravir-based vs. efavirenz-based treatment arms did not differ on baseline levels of plasma HCV RNA, HIV RNA, CD4^+^ T cell count, AFP, ALT, AST, or Fibroscan (ps > 0.14, effect sizes < 0.02), but did differ on hepatotoxicity (see Supplementary Tables 2, 3).

### Baseline Neurocognitive Comparisons

Neurocognitive test performances are summarized in Table 2. Results from the MANCOVA revealed a significant multivariate effect of group status for the motor domain (Wilks' Lambda = 0.92, *F* (6, 446) = 2.99, *p* < 0.01). Follow-up univariate analyses revealed a main effect on timed gait (*p* = 0.004), with significantly lower performance among the HIV-HCV co-infected (*p* = 0.006) and HIV mono-infected (*p* = 0.03) group compared to the uninfected controls. No other multivariate comparisons were statistically significant. Follow-up univariate analyses revealed a significant effect of group status on HVLT-R delay (*p* < 0.05). *Post-hoc* analyses using Tukey's test indicated that the HIV-HCV co-infected group performed more poorly than the uninfected controls (*p* = 0.03). Additionally, a main effect of group status was observed on Action Fluency (*p* < 0.01), with the HIV-HCV co-infected group achieving lower scores than the HIV mono-infected (*p* = 0.004) and the uninfected control group (*p* = 0.05). Supplementary Figures 1–4 provide boxplots of neurocognitive test raw scores by group.

**Table 2:** Baseline neurocognitive performance.

**Domain/test**	**HIV-HCV mean (SD)**	**HIVmean (SD)**	**Controls mean (SD)**	***F***	***p*-Value**	**Eta^**2**^**
**Psychomotor**				1.02	0.39	0.009
Digit Symbol	45 (15)	46 (16)	42 (16)	1.31	0.27	0.01
Color Trails 1	45 (15)	45 (14)	49 (16)	1.73	0.18	0.02
**Executive function**				*1.92*	*0.06*	*0.03*
Digits backward	7 (2)	8 (3)	7 (2)	1.67	0.19	0.01
Color trails 2	94 (30)	91 (31)	96 (28)	0.52	0.60	0.005
Action fluency	**10 (4)**	**12 (5)**	**11 (4)**	**5.52**	**0.005**	**0.05**
First name fluency	20 (5)	21 (5)	21 (4)	2.33	0.10	0.02
**Learning and memory**				1.70	0.10	0.02
HVLT-R learning	*22 (5)*	*24 (6)*	*24 (5)*	*2.97*	*0.05*	*0.03*
HVLT-R delay	**8 (2)**	**8 (3)**	**9 (2)**	**3.09**	**0.04**	**0.03**
BVMT-R learning	22 (7)	24 (7)	23 (7)	1.31	0.27	0.01
BVMT-R delay	9 (3)	9 (3)	9 (2)	0.77	0.57	0.005
**Motor**				**2.96**	**0.007**	**0.04**
Pegboard dom	66 (10)	64 (12)	64 (14)	1.08	0.34	0.009
Pegboard nondom	73 (15)	70 (14)	73 (16)	1.69	0.19	0.02
Timed gait	**16 (4)**	**16 (4)**	**14 (3)**	**5.60**	**0.004**	**0.05**

*Bold values indicate statistically significant differences between groups on that outcome variable. Italics indicate trend level significant difference between HIV groups on a given variable. HVLT-R, Hopkins Verbal Learning Test-Revised; BVMT-R, Brief Visuospatial Memory Test- Revised*.

At baseline, the rates of neurocognitive impairment in each domain (see Table 3) were as follows: *Psychomotor:* co-infected (9%), HIV mono-infected (8%), and uninfected controls (16%). Low performance on Color Trails 1 accounted for the higher rate of impairment among the healthy controls, but the maximum difference between these groups did not exceed one-half standard deviation. *Executive Function*: co-infected (20%), HIV mono-infected (16%), and uninfected controls (17%). Performance on Action Fluency differed most significantly by group (co-infected, mono-infected, controls: 30%, 10%, 16%, respectively). *Learning and Memory:* co-infected (19%), HIV mono-infected (21%), and uninfected controls (16%). *Motor:* co-infected (24%), HIV mono-infected (20%), and uninfected controls (10%).

**Table 3:** Baseline percentages of neurocognitive impairment by group.

	**Psychomotor speed**	**Executive function**	**Learning and memory**	**Motor**
HIV-HCV	9%	20%	19%	24%
HIV	8%	16%	21%	20%
Controls	16%	17%	16%	10%

*Impairment was defined by scoring ≤1.00 standard deviation below the mean*.

### Longitudinal Analyses

All HIV-HCV participants achieved undetectable HIV status by week 48. Viral suppression was maintained from week 48 to week 72 with no treatment failures by treatment arm. As reported previously, individuals randomized to efavirenz-based ART exhibited a higher degree of hepatotoxicity and number of adverse events compared to individuals randomized to raltegravir-based ART, but virological suppression did not differ by treatment regimen (20). Results indicated that HCV RNA viral load values did not differ significantly over time between treatment arms (Supplementary Table 3). As seen in Supplementary Table 2, analyses revealed that liver functioning as measured by Fibroscan, AFP, and AST did not differ significantly over time as a function of treatment arm (RAL vs. EFV). In contrast, ALT values differed significantly over time as a function of treatment arm (*p* < 0.01, eta^2^ = 0.12). Specifically, participants in the RAL treatment group had lower (healthier) AST values over time compared to the EFV group. Additional analyses using RM-ANOVA with FDR showed no significant difference between treatment arm over time on depression scores or CD4^+^ T cell count.

Neurocognitive performances improved on all measures except Color Trails 1 (*p* = 0.06) and Digit Span Backwards (*p* = 0.77). The largest gain in performance occurred between weeks 0 and 48. Repeated measures analyses revealed no significant effect of treatment arm and no interaction between treatment arm and time on any of the neurocognitive measures (Table 4). The frequency of neurocognitive impairment at week 72 was significantly lower than the frequency of impairment at baseline. Rates of impairment at week 72 were as follows for the HIV-HCV co-infected group: *Psychomotor* (7%), *Executive Function* (8%), *Learning and Memory* (3%), and *Motor* (9%). The rate of impairment at week 72 did not differ between treatment arms.

**Table 4:** Change in neurocognitive performance among HIV-HCV participants.

**Domain/tests**	***F***	***p*-Value**	**Effect size Eta^**2**^**
**Psychomotor**			
Digit symbol			
Treatment arm	1.78	0.19	0.03
Time	11.51	**<0.01**	0.35
Interaction	0.29	0.83	0.01
Color trails 1			
Treatment arm	3.03	0.09	0.05
Time	2.84	0.06	0.12
Interaction	0.27	0.85	0.01
**Executive function**			
Digits backward			
Treatment arm	0.001	0.97	0.001
Time	0.37	0.77	0.02
Interaction	0.86	0.47	0.04
Color trails 2			
Treatment arm	3.35	*0.07*	0.05
Time	19.41	**<0.01**	0.48
Interaction	0.88	0.46	0.04
Action fluency			
Treatment arm	0.70	0.41	0.01
Weeks	9.60	**<0.001**	0.31
Interaction	2.63	0.06	0.11
First Name fluency			
Treatment arm	0.01	0.91	0.001
Weeks	5.63	**0.002**	0.21
Interaction	0.48	0.70	0.02
**Learning and Memory**			
HVLT-R learning			
Treatment arm	0.14	0.71	0.002
Time	29.10	**<0.01**	0.58
Interaction	0.13	0.94	0.002
HVLT-R delay			
Treatment arm	0.71	0.40	0.01
Time		**<0.01**	0.54
Interaction	24.200.36	0.78	0.02
BVMT-R learning			
Treatment arm	0.81	0.37	0.01
Time	16.46	**<0.01**	0.44
Interaction	0.40	0.75	0.02
BVMT-R delay			
Treatment arm	0.81	0.37	0.01
Time	8.14	**<0.01**	0.28
Interaction	0.14	0.93	0.007
**Motor**			
Pegboard dominant			
Treatment arm	3.22	*0.07*	0.05
Time	31.38	**<0.001**	0.60
Interaction	0.82	0.49	0.04
Pegboard nondominant			
Treatment arm	3.15	0.08	0.05
Time	5.25	**0.003**	0.20
Interaction	1.19	0.32	0.05
Timed gait			
Treatment arm	0.07	0.80	0.001
Time	7.68	**<0.001**	0.27
Interaction	2.10	0.11	0.09

*Significant effects are in bold. Trend level effects p < 0.07 are indicated in italics*.

**Figure 1:**
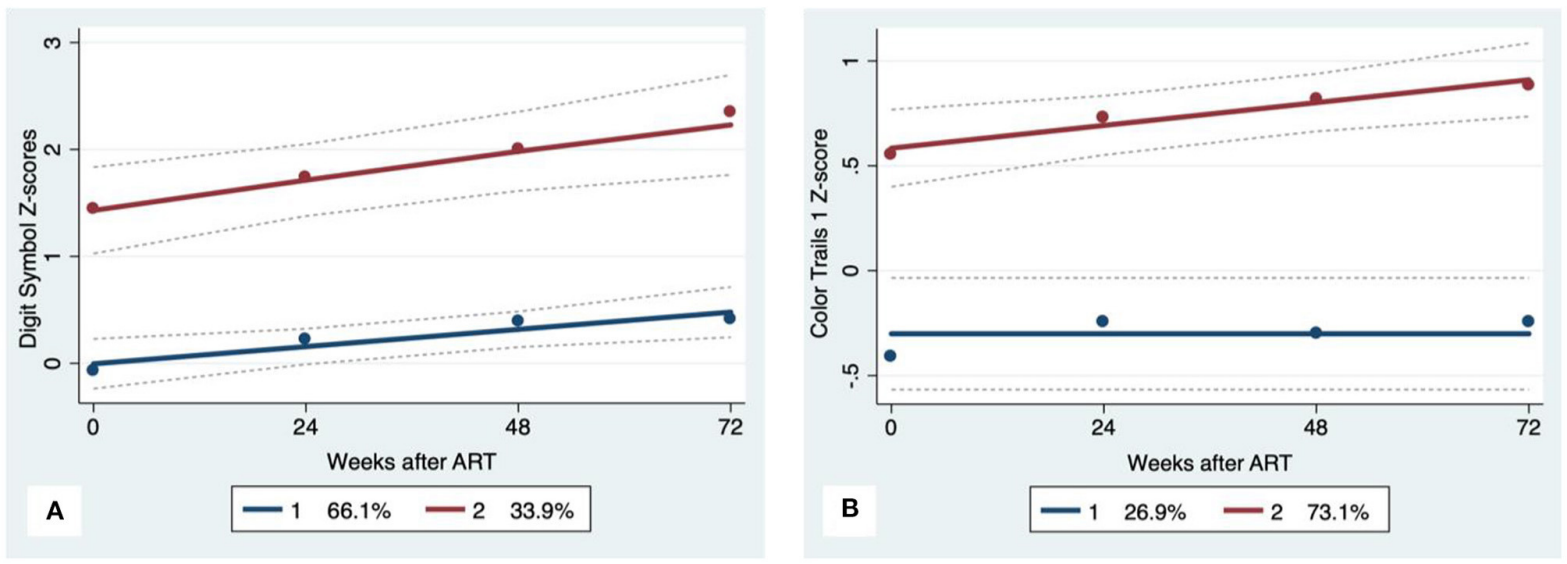
Psychomotor trajectory groups. Solid lines represent estimated trajectories, dot symbols are observed group means at each assessment wave, and dashed lines are 95% pointwise confidence intervals on the estimated trajectories. **(A)** WAIS-III Digit Symbol and **(B)** Color Trails 1.

**Figure 2:**
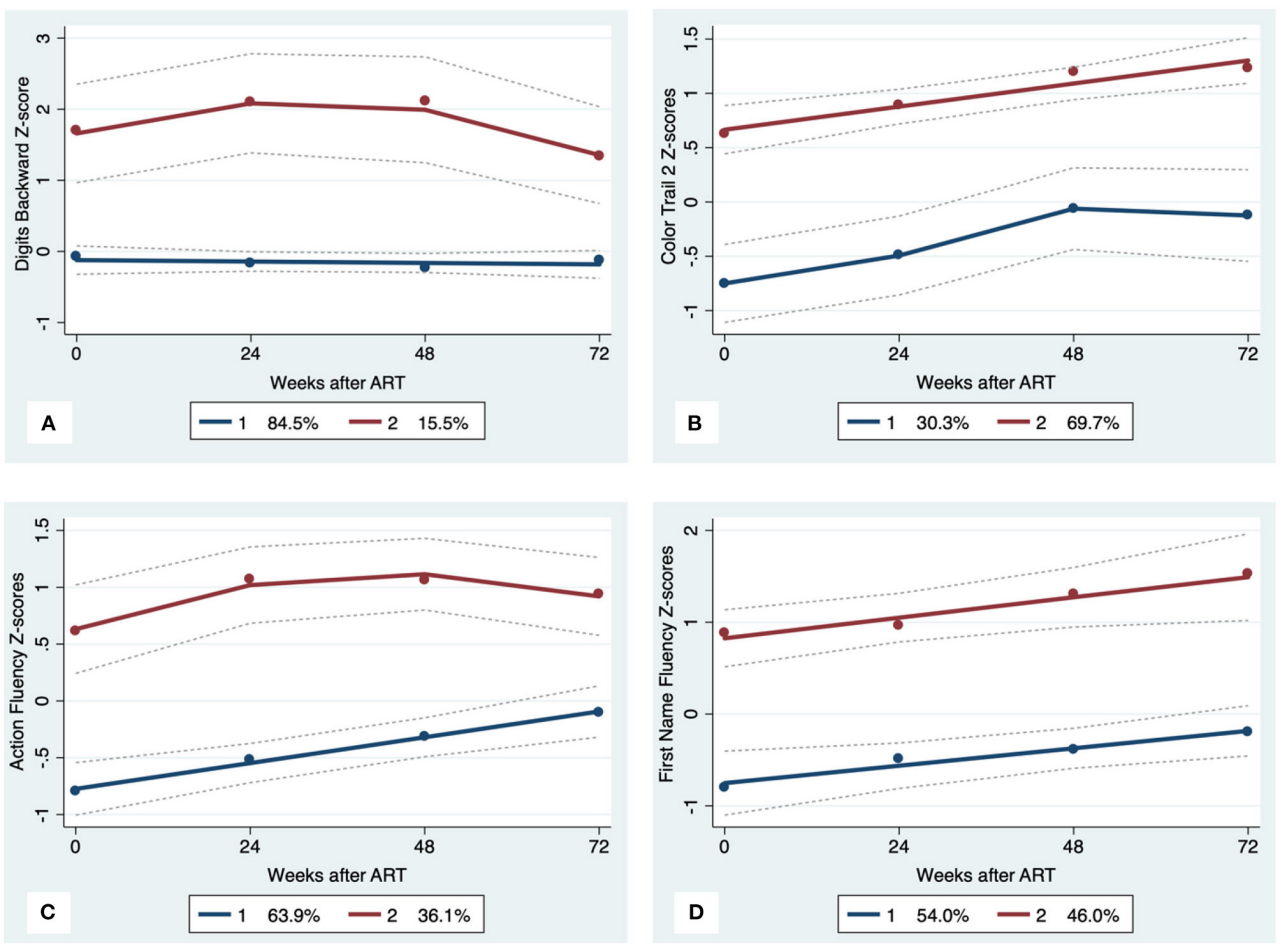
Executive Function trajectory subgroups, Solid lines represent estimated trajectories, dot symbols are observed group means at each assessment wave, and dashed lines are 95% pointwise confidence intervals on the estimated trajectories. **(A)** WAIS-III Digits Backward; **(B)** Color Trails 2; **(C)** Action Fluency; and **(D)** First Name Fluency.

**Figure 3:**
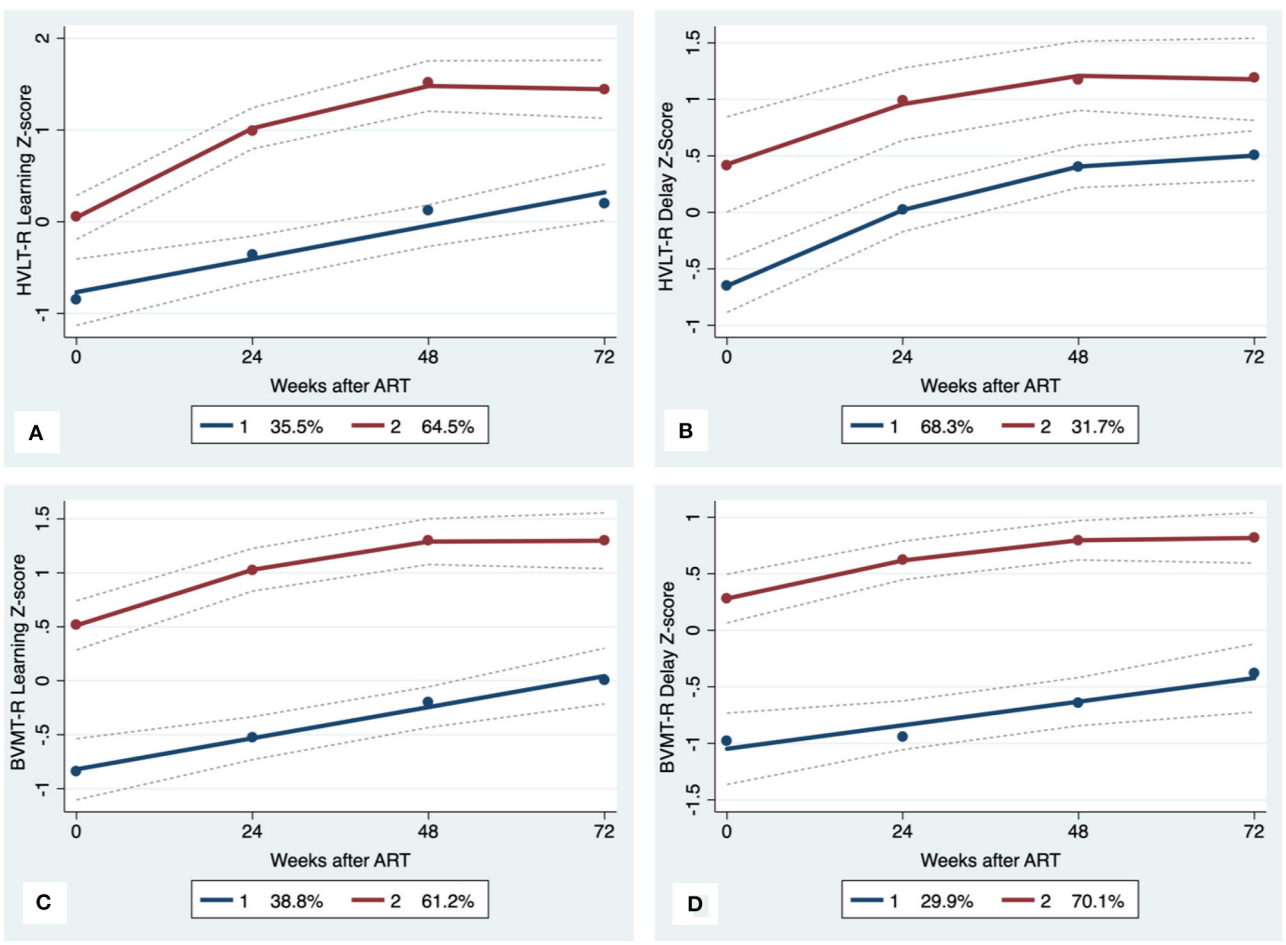
Learning and memory trajectory groups. Solid lines represent estimated trajectories, dot symbols are observed group means at each assessment wave, and dashed lines are 95% pointwise confidence intervals on the estimated trajectories. **(A)** HVLT-R Learning; **(B)** HVLT-R Delay; **(C)** BVMT-R Learning; and **(D)** BVMT-R Delay.

**Figure 4:**
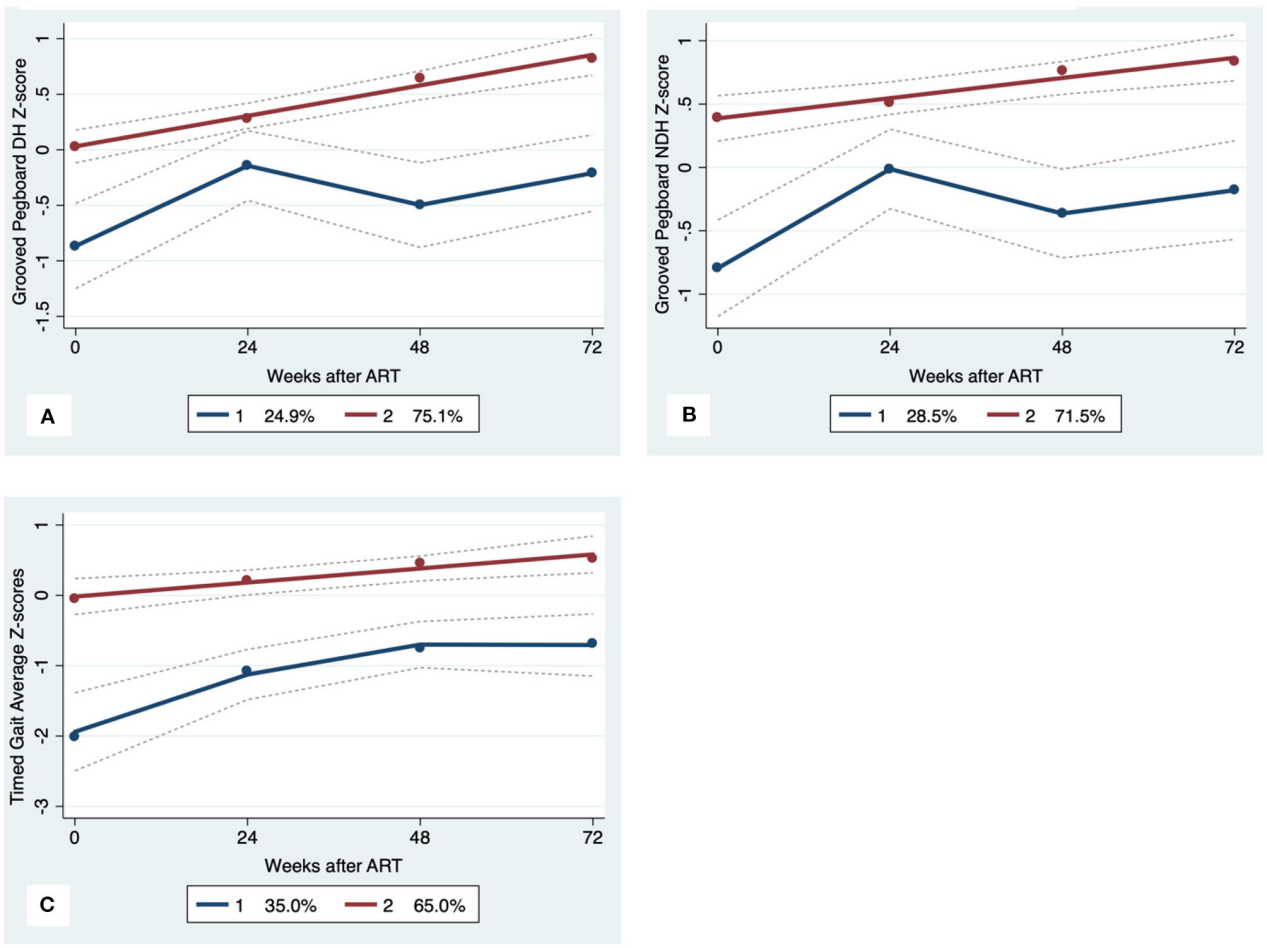
Motor Trajectory subgroups, Solid lines represent estimated trajectories, dot symbols are observed group means at each assessment wave, and dashed lines are 95% pointwise confidence intervals on the estimated trajectories. **(A)** Grooved Pegboard Dominant Hand, **(B)** Grooved Pegboard Non-Dominant Hand; and **(C)** Timed Gait.

GBTA modeled from enrollment to week 72 revealed that the best fitting models for all neurocognitive tests were obtained when using two latent trajectory groups for modeling (see Supplementary Tables 4–7). The 2-group latent class trajectories represent HIV-HCV participant clusters defined by higher vs. lower neurocognitive performance over time (minimum threshold requirement for group assignment ≥0.7 for posterior probability and/or ≥4.0 for odds ratio). Closer examination of the trajectories for each neurocognitive test revealed that, in general, both subgroups demonstrated improved performances over time (Figures 1–4). However, the low performing trajectory group remained impaired on the timed gait task at week 72 (Figure 4C). Results indicated that treatment status predicted latent class trajectories for a single neurocognitive test, Action Fluency. Participants in the RAL treatment arm were approximately four times as likely as participants in the EFV treatment arm to be in the low scoring latent trajectory, OR = 3.82, CI:1.3–11.4 (Figure 2C). Baseline demographic variables (i.e., age, sex, or education) were unrelated to trajectory subgroups on any of the individual neurocognitive tests. Regression analyses revealed that baseline lower plasma CD4^+^ T cell count and plasma viral load predicted membership in the low trajectory group on the timed gait task (variables examined included those listed in Table 1).

## Discussion

Our study is the first prospective, multi-dimensional investigation of neurocognitive outcomes among PLWH with co-occurring HCV infection before and after *de novo* ART. Consistent with prior studies (37–39), HIV-HCV co-infected individuals exhibited worse neurocognitive performance at baseline compared to HIV mono-infected individuals and healthy uninfected controls. Additionally, a larger proportion of co-infected individuals met clinical criteria for neurocognitive impairment prior to ART use. Neurocognitive performance improved within 48 weeks of ART onset, but latent trajectory analyses identified a subgroup of HIV-HCV co-infected individuals who demonstrated persistent impairment in the motor domain. Results of this study emphasize the need to investigate variability that exists within groups as a path toward discovery of driving mechanisms. In this study, we utilized group-based trajectory modeling to identify latent phenotypes of higher vs. lower neurocognitive performance. This approach revealed clusters of individuals that were predicted at baseline by differences in CD4^+^ T cell count and HIV viral load.

Cross-sectional studies report increased severity of neurocognitive complications among PLWH with co-occurring HCV infection. Our study results align with previously reported outcomes in that significant neurocognitive sequelae are prevalent with HIV-HCV co-infection. HCV may potentiate viral-host mechanisms (e.g., monocyte/macrophage activation) of HIV before and after ART initiation. The increased impact of HIV co-infection on brain systems may explain the prevalence of chronic health complications (e.g., frailty) reported in HCV co-infected PLWH receiving ART despite achieving sustained HIV viral suppression (40, 41). Baseline comparisons revealed more severe and clinically relevant neurocognitive symptoms among individuals with co-infection. After controlling for depressive symptoms, PLWH with co-occurring HCV infection exhibited worse performance on measures of motor function and select measures of executive function.

Longitudinal analyses revealed significant gains in neurocognitive performance over 72 weeks of effective ART, with the largest gains observed in the first 48 weeks. The magnitude of change exceeded chance and the slope of improvement exceeded the window in which practice effects from repeat testing are most pronounced. Critically, the magnitude of change in neurocognitive performance was clinically meaningful, as the rate of neurocognitive impairment at week 72 was <10% for each neurocognitive domain. These results confirm hypotheses from prior cross-sectional work (11) suggesting that brain dysfunction associated with co-infection is manifest in the setting of uncontrolled HIV viremia.

Latent models identified distinct neurocognitive phenotypes (higher vs. lower cognitive performance subgroups) that followed and maintained separate trajectories throughout the course of this study. To our knowledge, this is the first study application of latent trajectory modeling to identify subgroups of HIV-HCV co-infection. Results from the GBTA revealed three important findings. First, while the subgroups differed on the degree of neurocognitive complications, both clusters followed a positive trajectory after ART, consistent with improved performance in most domains. Second, baseline neurocognitive status was predictive of trajectory subgroup designation. That is, the low trajectory group demonstrated worse baseline neurocognitive performance. Similar results have been described in prior studies of HIV mono-infection before and after the start of suppressive ART (42). Finally, more than one-third of the co-infected group exhibited persistent impairment in motor function despite otherwise successful ART.

The impaired motor performance among individuals with higher HIV viral load and lower plasma CD4^+^ T cell count at baseline may reflect an early signature of chronic brain dysfunction in co-infected individuals. HCV co-infection is a known predictor of frailty in PLWH receiving suppressive ART (10, 40, 42, 43). Our recent work using machine learning and multi-modal neuroimaging identified evidence of visuomotor dysfunction among PLWH who met clinical criteria for frailty (44). Additional work is needed to define putative causal pathways between HCV co-infection, motor impairment, and frailty in PLWH. Additionally, interventions aimed at supporting motor function at the time of HIV treatment onset may improve long-term clinical outcomes in HIV-HCV co-infected adults.

Results reported in this study represent the first prospective investigation of neurocognitive performance before and after *de novo* ART in a large sample of HIV-HCV co-infected individuals. Additional strengths of the current study include the use of data driven algorithms to examine neurocognitive performance adjusted for demographic variables relevant to the local population. This approach overcomes the limitations of traditional methods that require large samples to stratify data based on assumptions as to how specific factors (e.g., sex, age) impact neurocognitive status. Regression-based norms utilize a quantitative approach to adjust neurocognitive performances for psychosocial factors, which provides a more accurate estimate of brain structure and function. Additional strengths include the comprehensive approach to neurocognitive assessment and use of latent trajectory modeling. Limitations include the absence of comparison groups for longitudinal analysis and the absence of neuroimaging. Additionally, our sample was predominately male, and therefore we did not have sufficient power to examine sex-related differences in outcomes. Longitudinal studies of HIV mono-infected, HCV mono-infected, and uninfected controls are needed, including investigations that incorporate multi-modal neuroimaging. Latent analytic models are encouraged to confirm the relevance of early and persistent motor dysfunction in adults with HIV-HCV co-infection.

## Data Availability Statement

The raw data supporting the conclusions of this article will be made available by the authors, without undue reservation.

## Ethics Statement

The studies involving human participants were reviewed and approved by Vietnam Ministry of Health Hospital for Tropical Diseases (HTD) in Ho Chi Minh City (HCMC), University of Hawaii at Manoa in the U.S., and Oxford University Tropical Research Ethics Committee in the U.K. The patients/participants provided their written informed consent to participate in this study.

## Author Contributions

RP, CS, LN, DC, GC, TP, and VL contributed to conception and design of the study. NC, NT, and VL were responsible for study data collection and interpretation. AB and JB completed data analysis for the proposed manuscript. RP, CS, LN, AB, DC, GC, TP, JM, JB, and TL contributed to drafting the manuscript. TL contributed to conception and design of the study. All authors contributed to manuscript revision and read and approved the submitted version.

## Conflict of Interest

The authors declare that the research was conducted in the absence of any commercial or financial relationships that could be construed as a potential conflict of interest.
