# Supplementary material for: Neurocognitive Trajectories After 72 Weeks of First-Line Anti-retroviral Therapy in Vietnamese Adults With HIV-HCV Co-infection
Source: Front Neurol. 2021 Mar 12;12:602263. doi: 10.3389/fneur.2021.602263 (PMC7996090; doi:10.3389/fneur.2021.602263)
Supplement: Supplementary file 1 [file Table_1.docx]

| **Supplementary Table 1.** Description of neurocognitive tests grouped by domain | | |
| --- | --- | --- |
| **Domain functioning** | **Test name** | **Measurement** |
| Learning and Memory | HVLT-R | Total words recalled over learning trials |
|  | HVLT-R | Total words recalled on delayed trial |
|  | BVMT-R | Total figures over 3 learning trials |
|  | BVMT-R | Total figures on delayed trial |
| Psychomotor | Digit Symbol (WAIS-IV) | Number correct |
|  | Color Trail 1 | Time to completion (seconds) |
| Executive Function | Digit Span Backward (WAIS-IV) | Number of correct |
|  | Color Trail 2 | Time to completion (seconds) |
|  | Action Fluency | Number correct |
|  | First Name Fluency | Number correct |
| Gross motor | Grooved Pegboard (Dominant) | Time to complete dominant hand (seconds) |
|  | Grooved Pegboard (non-dominant) | Time to complete non-dominant hand (seconds) |
|  | Timed Gait | Average time across three trials (seconds) |

| **Supplementary Table 2.** Means and standard deviations for liver parameters over time by treatment arm within HIV/HCV group | | | | | | | | | | | | |
| --- | --- | --- | --- | --- | --- | --- | --- | --- | --- | --- | --- | --- |
|  | **RAL** | | | | | **EFV** | | | | | **Effect Sizes in partial eta^2^** | |
|  | *0* | *24* | *48* | *72* | *n* | *0* | *24* | *48* | *72* | *n* | *Time* | *Time x Treatment* |
| Fibroscan (kPA) | 10(9) | n/a | 9(7) | 9(7) | 29 | 9(8) | n/a | 8(5) | 8(6) | 32 | .09 | .01 |
| AFP (mg/mL) | 20(84) | 11(31) | 6(13) | 5(7) | 33 | 8(24) | 5(4) | 6(9) | 6(10) | 36 | .05 | .06 |
| ALT (U/L) | 37(17) | 58(49) | 63(53) | 47(28) | 33 | 40(19) | 79(78) | 72(35) | 75(42) | 36 | **.37** | **.12** |
| AST (U/L) | 42(15) | 55(38) | 51(34) | 41(18) | 33 | 46(16) | 65(47) | 58(24) | 58(29) | 36 | **.19** | .08 |
| RAL=Raltegravir, EFV=Efavirenz, n=subgroup sample size for longitudinal analysis, Bold values indicate p <. 01. | | | | | | | | | | | | |

| **Supplementary Table 3.** HCV RNA viral load from baseline to week 72 in HIV/HCV co-infected subgroup by treatment arm | | | | | | |
| --- | --- | --- | --- | --- | --- | --- |
|  | **RAL arm** | **n** | **EFV arm** | **n** | ***p*-value** | **Effect size** |
| Week 0 HCV RNA VL: M(IQR) | 2350(48-7715) | 39 | 10400(826-16500) | 41 | .87 | .03 |
| Week 24 HCV RNA VL: M(IQR) | .05(.04-.23) | 19 | .12(.06-.35) | 15 | .19 | .02 |
| Week 48 HCV RNA VL: M(IQR) | .06(.05-.32) | 11 | .29(.05-.38) | 13 | .46 | .04 |
| Week 72 HCV RNA VL: M(IQR) | .13(.09-.21) | 9 | .12(.05-.21) | 11 | .55 | .04 |
| *Note.* M=Median, IQR=Interquartile range. Mann-Whitney U Tests were conducted to compare median values between treatment groups at each assessment week. | | | | | | |

| **Supplementary Table 4.** Parameter estimates for psychomotor domain latent class trajectories | | | | |
| --- | --- | --- | --- | --- |
|  | **Digit Symbol** | | **Color Trails 1** | |
| Trajectory Group | *Low* | *High* | *Low* | *High* |
| Group Size: n | 48 | 22 | 18 | 52 |
| AvePP | .95 | .97 | .90 | .95 |
| OCC | 8.7 | 66.8 | 25.8 | 6.4 |
| TotProb | 66.1 | 33.9 | 26.9 | 73.1 |
| RAL subgroup: n | 21 | 12 | 6 | 27 |
| EFV subgroup: n | 27 | 10 | 12 | 25 |
| AvePP=Average posterior probability value (i.e., good model criteria > .70 for each group), OCC=Odds of correct classification for trajectory group (i.e., criteria for good model OCC > 5.0 for each group), TotProb=Proportion assigned to group according to maximum posterior probability assignment rule, RAL=Raltegravir, EFV=Efavirenz. | | | | |

| **Supplementary Table 5.** GBTA parameter estimates for executive function domain latent class trajectories | | | | | | | | |
| --- | --- | --- | --- | --- | --- | --- | --- | --- |
|  | **Digit Span (B)** | | **Color Trail 2** | | **Action Fluency** | | **Name Fluency** | |
| Trajectory Group | *Low* | *High* | *Low* | *High* | *Low* | *High* | *Low* | *High* |
| Group Size: n | 60 | 10 | 20 | 50 | 47 | 23 | 38 | 32 |
| AvePP | .98 | .98 | .92 | .96 | .93 | .96 | .94 | .93 |
| OCC | 9.1 | 326.8 | 30.4 | 8.6 | 6.5 | 45.8 | 12.2 | 15.7 |
| TotProb | 84.4 | 15.6 | 29.6 | 70.4 | 63.9 | 36.1 | 54.0 | 46.0 |
| RAL subgroup: n | 29 | 4 | 9 | 24 | 27 | 6 | 19 | 14 |
| EFV subgroup: n | 31 | 6 | 11 | 26 | 20 | 17 | 19 | 18 |
| AvePP=Average posterior probability value (i.e., good model criteria > .70 for each group), OCC=Odds of correct classification for trajectory group (i.e., criteria for good model OCC > 5.0 for each group), TotProb=Proportion assigned to group according to maximum posterior probability assignment rule, RAL=Raltegravir, EFV=Efavirenz, (B)=Backwards. | | | | | | | | |

| **Supplementary Table 6.** GBTA parameter estimates for learning and memory domain latent class trajectories | | | | | | | | |
| --- | --- | --- | --- | --- | --- | --- | --- | --- |
|  | **HVLT-R Total** | | **HVLT-R Delay** | | **BVMT Total** | | **BVMT Delay** | |
| Trajectory Group | *Low* | *High* | *Low* | *High* | *Low* | *High* | *Low* | *High* |
| Group Size: n | 26 | 44 | 49 | 21 | 27 | 43 | 21 | 49 |
| AvePP | .89 | .96 | .91 | .85 | .95 | .96 | .94 | .97 |
| OCC | 13.4 | 14.2 | 4.5 | 14.1 | 28.0 | 15.9 | 34.4 | 16.5 |
| TotProb | 35.5 | 64.5 | 68.3 | 31.7 | 38.8 | 61.2 | 29.9 | 70.1 |
| RAL subgroup: n | 16 | 17 | 25 | 8 | 13 | 20 | 10 | 23 |
| EFV subgroup: n | 10 | 27 | 24 | 13 | 14 | 23 | 11 | 26 |
| AvePP=Average posterior probability value (i.e., good model criteria > .70 for each group), OCC=Odds of correct classification for trajectory group (i.e., criteria for good model OCC > 5.0 for each group), TotProb=Proportion assigned to group according to maximum posterior probability assignment rule, RAL=Raltegravir, EFV=Efavirenz. | | | | | | | | |

| **Supplementary Table 7.** GBTA parameter estimates for motor function domain latent class trajectories | | | | | | |
| --- | --- | --- | --- | --- | --- | --- |
|  | **Grooved Pegboard (D)** | | **Grooved Pegboard (ND)** | | **Timed Gait Average** | |
| Trajectory Group | *Low* | *High* | *Low* | *High* | *Low* | *High* |
| Group Size: n | 17 | 53 | 16 | 54 | 24 | 46 |
| AvePP | .84 | .94 | .87 | .96 | .92 | .95 |
| OCC | 16.5 | 5.3 | 22.3 | 7.0 | 23.6 | 9.8 |
| TotProb | 24.7 | 75.3 | 23.0 | 77.0 | 35.0 | 65.0 |
| RAL subgroup: n | 6 | 27 | 4 | 29 | 12 | 21 |
| EFV subgroup: n | 11 | 26 | 12 | 25 | 12 | 25 |
| AvePP=Average posterior probability value (i.e., good model criteria > .70 for each group), OCC=Odds of correct classification for trajectory group (i.e., criteria for good model OCC > 5.0 for each group), TotProb=Proportion assigned to group according to maximum posterior probability assignment rule, RAL=Raltegravir, EFV=Efavirenz, (D)=Dominant hand, (ND)=Nondominant hand. | | | | | | |
