# Supplementary figures and images for: Neurocognitive Trajectories After 72 Weeks of First-Line Anti-retroviral Therapy in Vietnamese Adults With HIV-HCV Co-infection
Source: Front Neurol. 2021 Mar 12;12:602263. doi: 10.3389/fneur.2021.602263 (PMC7996090; doi:10.3389/fneur.2021.602263)

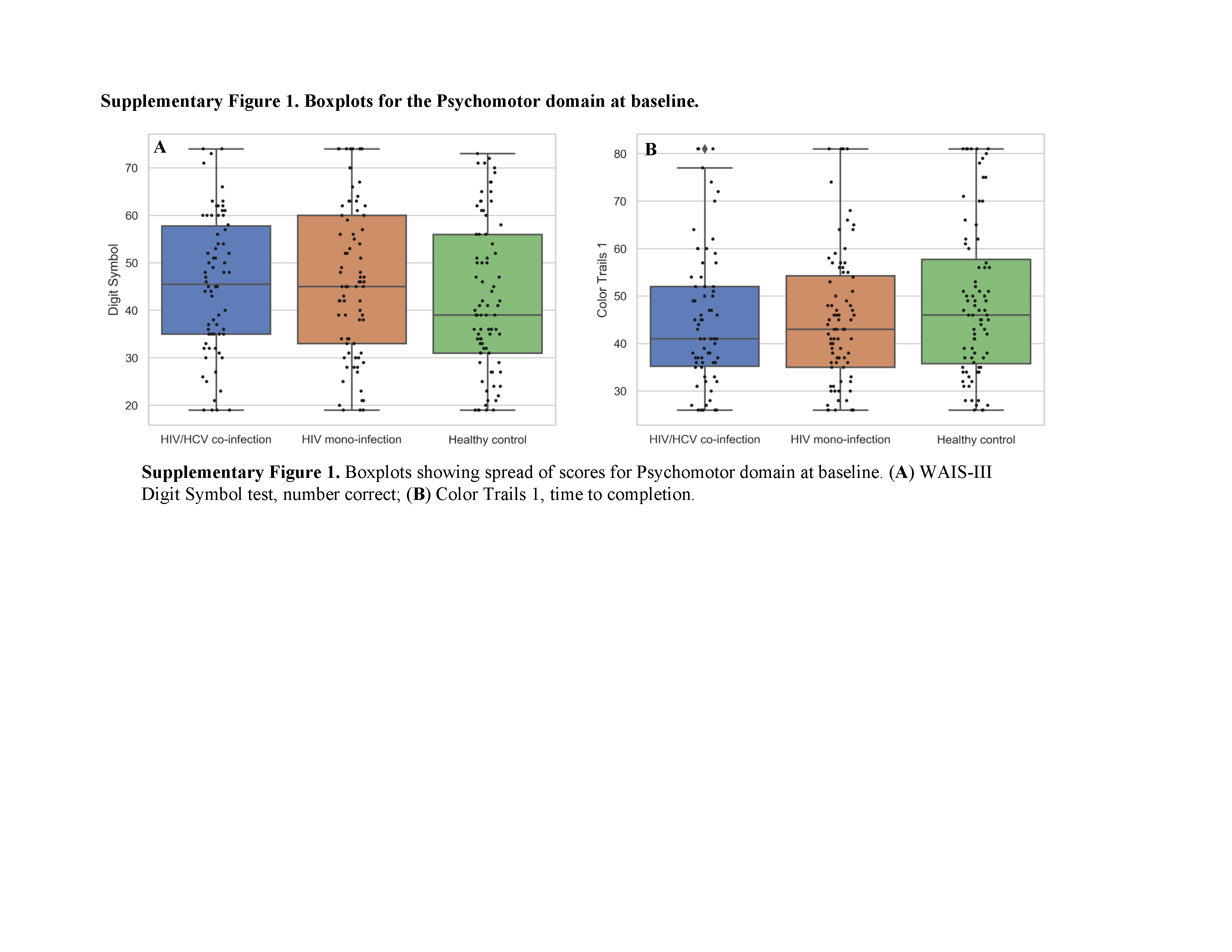

Supplement: Supplementary file 2 [file Image_1.TIFF]

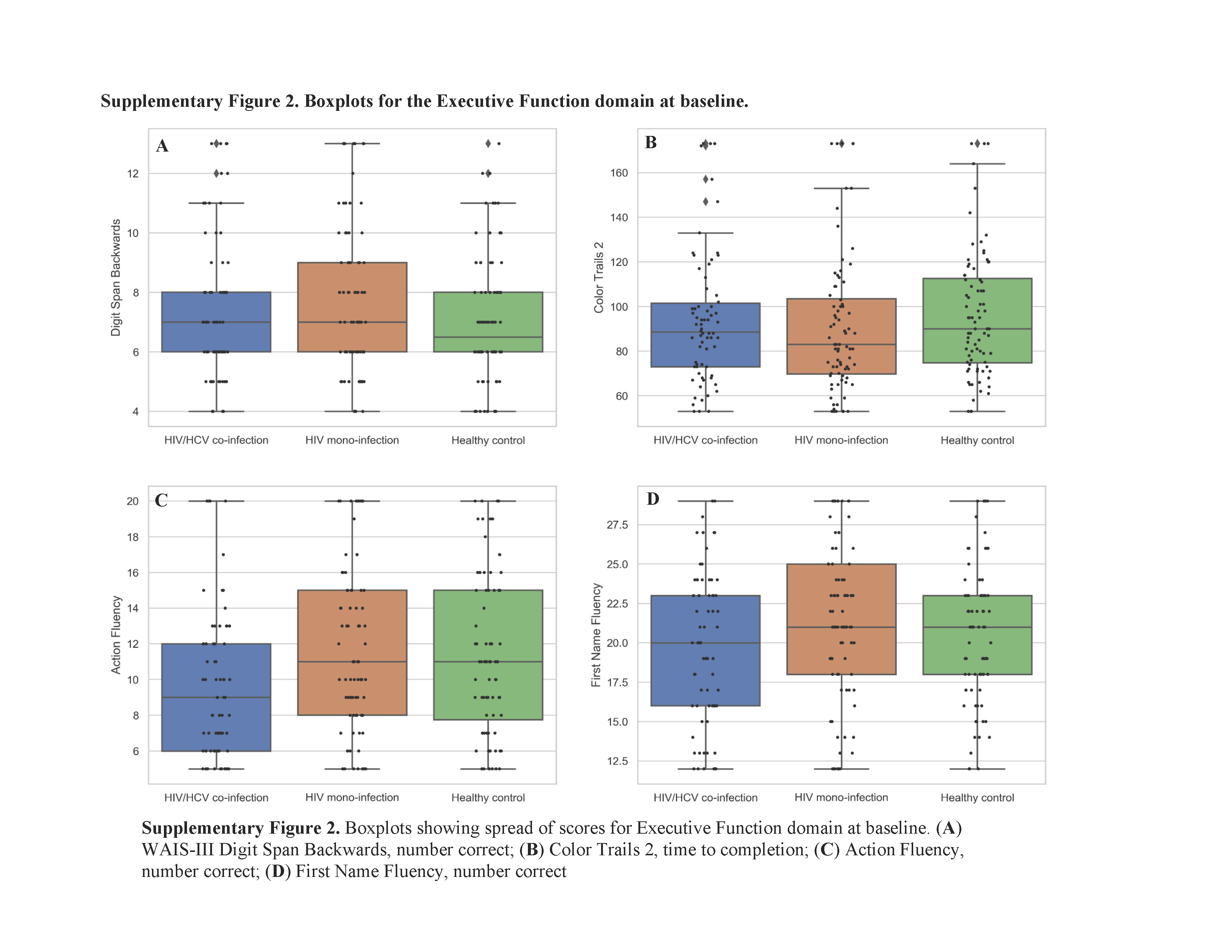

Supplement: Supplementary file 3 [file Image_2.TIFF]

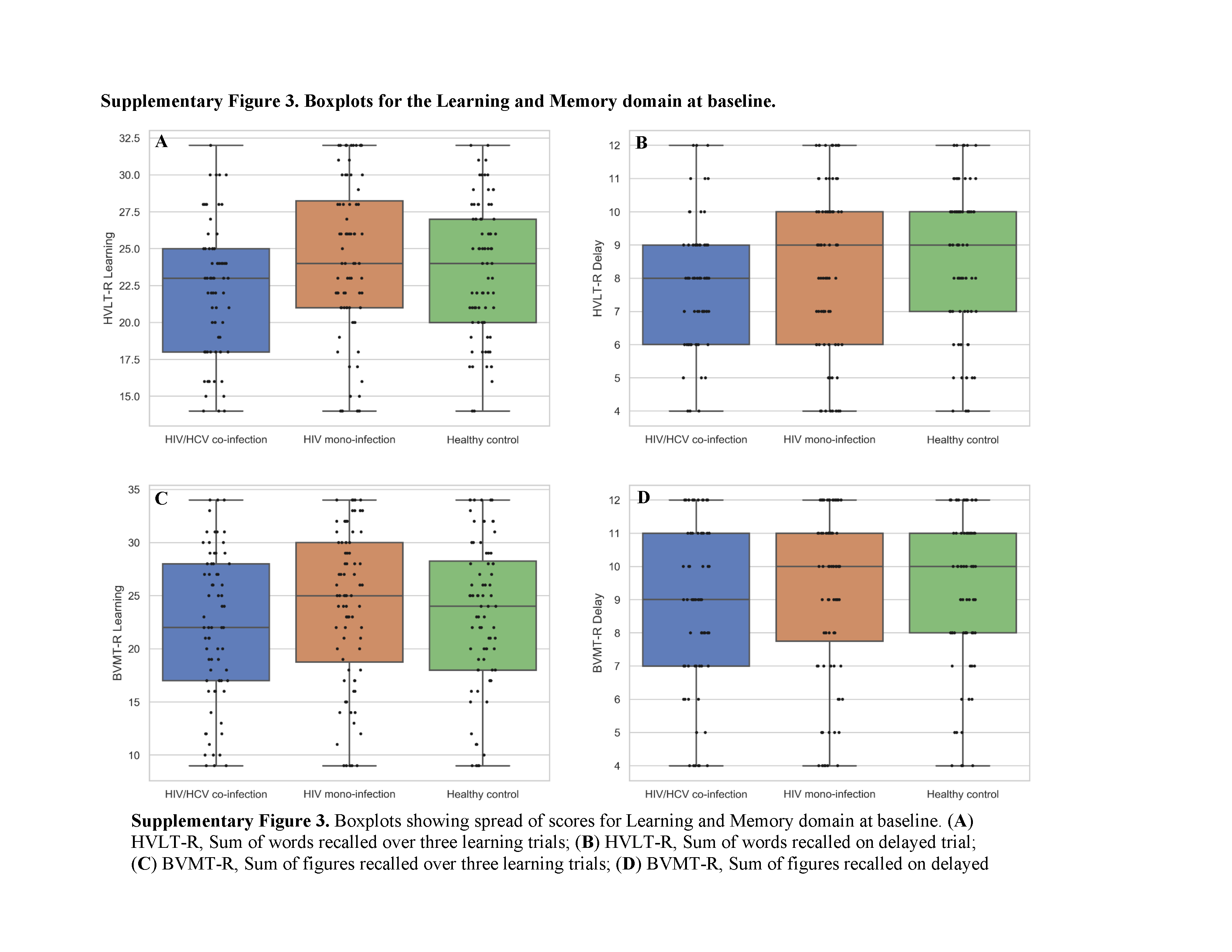

Supplement: Supplementary file 4 [file Image_3.TIFF]

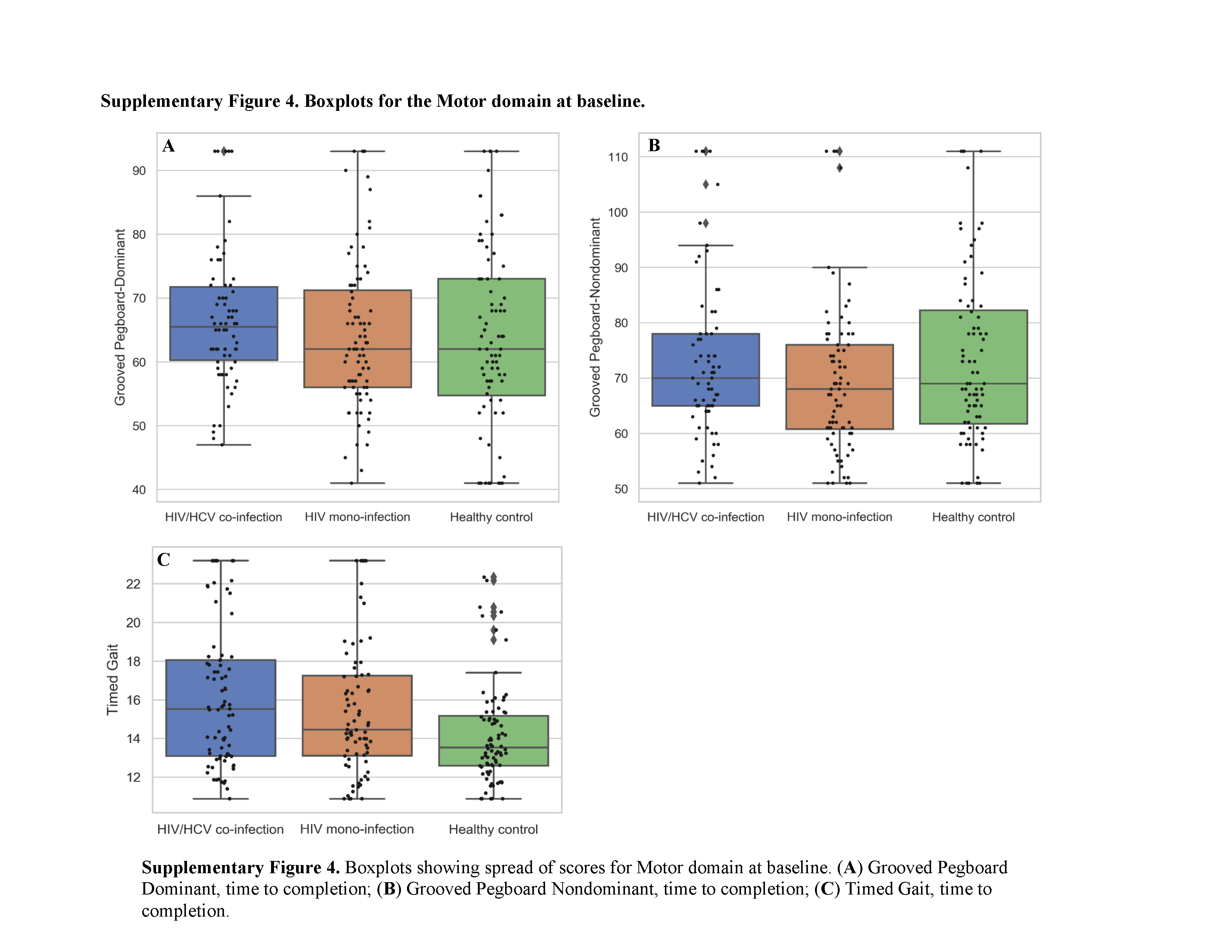

Supplement: Supplementary file 5 [file Image_4.TIFF]
